# Issues and missed opportunities in lymph node assessment after neoadjuvant chemotherapy

**DOI:** 10.1002/path.6479

**Published:** 2025-10-24

**Authors:** Lucy Ryan, Elena Provenzano, Anita Grigoriadis, Sarah E Pinder

**Affiliations:** ^1^ Cancer Bioinformatics, School of Cancer & Pharmaceutical Sciences, Faculty of Life Sciences and Medicine, King's College London London UK; ^2^ School of Cancer & Pharmaceutical Sciences, Faculty of Life Sciences and Medicine, King's College London London UK; ^3^ Cambridge University Hospitals NHS Foundation Trust Cambridge UK; ^4^ NIHR Cambridge Biomedical Research Centre Cambridge UK; ^5^ PharosAI, School of Cancer & Pharmaceutical Sciences, Faculty of Life Sciences and Medicine, King's College London London UK

**Keywords:** lymph nodes, neoadjuvant therapy response, breast cancer

## Abstract

Assessment of axillary lymph nodes in breast cancer patients following neoadjuvant chemotherapy (NACT) is a crucial part of the clinical and pathological assessment of the disease and has prognostic and management implications. This, however, currently lacks standardisation and focuses only on the number of lymph nodes with metastases still present, the largest metastasis, and the presence of pathological complete response. Potential changes in any residual disease or within the lymph node parenchyma are not examined. Novel methods of more nuanced approaches are rare in the literature, even when considering multiple cancer types, but can offer an insight into the potential additional information to be gained and improvement in patient stratification. Given how common NACT is as the backbone of cancer therapy, there is a surprising lack of research into the lymph node response and determination of the biological factors driving what is seen histologically. Furthermore, with NACT now being administered alongside immunotherapy, there is an increasing need to understand the functional and architectural changes induced in the lymph nodes by metastatic tumour and systemic therapies. This review summarises current approaches, with breast cancer as an exemplar, and discusses the literature investigating a possible more granular approach to lymph node assessment after NACT. Translating these multiple carcinoma studies to breast cancer patients may prompt tissue‐based research and, with clinical validation studies, changes to the reporting of lymph node response, for example percentage of viable tumour and immunological architectural features such as germinal centres. © 2025 The Author(s). *The Journal of Pathology* published by John Wiley & Sons Ltd on behalf of The Pathological Society of Great Britain and Ireland.

## Introduction

Neoadjuvant chemotherapy (NACT) regimens were historically recommended for patients with locally advanced or large breast carcinomas, the aim being to shrink the lesion and facilitate breast‐conserving surgery in those who would otherwise have inoperable disease or who would require a mastectomy. NACT is now used more widely for patients with a poorer prognosis (e.g. with lymph node metastasis), especially in those who are likely to have a good response to such treatment, particularly those with triple‐negative [absence of oestrogen receptor (ER), progesterone receptor (PR), and human epidermal growth factor receptor 2 (HER2) overexpression or gene amplification] or HER2‐positive disease [1]. The backbone of breast cancer patients’ NACT are taxanes, anthracyclines, and platinum‐based agents, along with anti‐HER2‐targeted therapy for those with HER2‐positive disease, and increasingly immunotherapy for those with triple‐negative breast cancer (TNBC). Furthermore, patients with residual disease after completion of NACT are at high risk of relapse and may subsequently be considered for adjuvant therapies (e.g. capecitabine, olaparib, and T‐DM1 as appropriate) [2]. The previous use of NACT informs patient selection for specific adjuvant therapies and the addition of these adjuvant therapies has resulted in improved patient outcomes [3].

Response to NACT in the breast and axilla is evaluated clinically through physical examination, usually undertaken by breast surgeons and by radiologists potentially using a range of imaging modalities. From this assessment, presurgical treatment efficacy is inferred. However, inherently, there is variation in physical examination skills and it is well recognised that magnetic resonance imaging (MRI) and ultrasound are both fallible in determining response of the tumour, and in the case of axillary ultrasound, lower sensitivity has been reported in patients with certain tumour characteristics: invasive lobular carcinoma, grade I/II, unifocal disease, and size less than 20 mm [4, 5, 6]. Within the lymph nodes after NACT, MRI sensitivity and specificity for detecting nodal metastases have been reported as 33.93% and 82.76%, respectively [7]. Pathological examination following surgery is key in determining the presence and amount of remaining invasive cancer. Aside from tumour cells, there are a variety of histopathological changes described at the primary tumour bed, with oedematous or fibrotic stroma and lipid‐ or haemosiderin‐laden macrophages being the most frequent [8, 9, 10]. Historically, although various definitions of pathological complete response (pCR) have been used, it is now accepted that complete eradication of invasive cancer in the breast and metastasis in the axilla is the most appropriate [11]. However, categorisation of the degree of any remaining invasive cancer is less unified. There is prognostic significance to the degree of residual carcinoma and, indeed, treatment decisions are based on this pathological assessment [2, 11, 12, 13, 14]. Nevertheless, local practices in reporting the extent of residual carcinoma vary depending on the preferences of the multidisciplinary team and treating oncologists.

The thoroughness of sampling and of histological examination, alongside the variation and duration of NACT agents received and, in particular, the inherent nature of the tumour biology, potentially explains the varying rates of pCR published. Hormone receptor (HR)‐negative/HER2‐positive invasive breast cancer and TNBC generally have the highest rates of pCR but ranges published vary between 0% and 60% [15, 16, 17, 18].

Most commonly, a single response score is given to cover both the primary site and the lymph nodes. In breast cancer, but also in other cancer types, assessing the lymph node component has tended to be less detailed. Few studies have looked solely at the rate of pCR in the axillary lymph nodes and reported it to be an independent prognostic factor [13, 19, 20, 21, 22, 23, 24]. A meta‐analysis found a lymph node pCR rate in HR‐negative/HER2‐positive breast cancer of 60% and in TNBC of 48% [25]. For comparison in the breast only, meta‐analysis of these two subtypes found pCR rates of 39% and 31% respectively, highlighting that potential differences in response may signify divergent tumour biology and differences in the microenvironment at the primary and lymph node sites [16].

A variety of methods have been applied to the assessment of therapeutic response or regression in lymph nodes. There is a lack of uniformity in this, and the most frequently used methods [such as tumour, node, metastasis (TNM) staging and residual cancer burden (RCB)] focus on descriptions of the number of lymph nodes with metastases still present, whilst the RCB in addition incorporates measurement of the largest metastasis; this leaves reporting pathologists with no requirements to consider the lymph nodes in greater depth [10, 14, 26].

This review discusses the current assessment of the axillary lymph nodes following NACT in breast cancer patients and addresses areas that potentially motivate considerations of a more comprehensive assessment.

## Current practice

All lymph nodes excised following NACT are examined for residual metastases with haematoxylin and eosin (H&E) staining according to The Royal College of Pathologists guidelines [27]. This includes examination of sentinel lymph nodes (in patients previously assessed to be node‐negative), in many centres with three H&E levels. Typically, axillary lymph node clearance specimens are sliced, and each lymph node is examined in its totality but with only one H&E section. If metastases are present, they are measured and categorised as isolated tumour cells (ITCs), micrometastases, or macrometastases, depending on the number of cells or size (200 cells/<0.2 mm versus 0.2 mm to 2 mm versus >2 versusmm respectively). Routine intraoperative assessment after NACT is problematic; one‐step nucleic acid amplification (OSNA) is not recommended as it is not calibrated for detection of isolated tumour cells and it does not allow comments to be made on fibrosis, whilst frozen section histological assessment is challenging [28, 29].

Cytokeratin immunohistochemistry can be used to confirm tumour cell presence in suspicious cases, with scanty evidence suggesting that AE1/AE3 is the most valuable marker [30]. It is likely, but there is very limited literature available, that such additional immunohistochemistry is more frequently utilised in samples after NACT [31]. In addition, the International Collaboration on Cancer Reporting (ICCR) strongly encourages, and some multidisciplinary teams (MDTs) ask for, reports to include information on the number of lymph nodes with no residual metastasis but showing complete response changes, when considering further radiotherapy treatment, in correlation with pretreatment radiology [32].

The lack of consensus in the reporting of lymph node response extends to the classification systems. Within breast cancer, the American Joint Committee on Cancer (AJCC) TNM eighth edition measures metastases’ size as the largest contiguous focus of metastatic tumour cells excluding intervening fibrosis, resulting in the downstaging of nodal disease in many cases. This is especially the case for the categorisation of ITCs, leading to inconsistencies in the classification of post‐treatment lymph node code (ypN) for these. Some pathologists unify the assessment through reporting the RCB in breast cancers after NACT [14]. The lymph node component of this algorithm includes the number of lymph nodes with metastases present and the maximum microscopic dimension of the largest metastasis, which is defined as the distance between tumour cells including fibrosis. The RCB classification, which is divided into four classes (0–3), has repeatedly shown prognostic value [33, 34, 35].

In addition to the number of involved nodes after NACT and description of the number of lymph nodes with complete response histologically, other features suggested to be of potential interest include the ratio of involved nodes to dissected nodes, the ratio of non‐responsive to responsive lymph nodes, and the ratio of lymph nodes with residual metastasis to the total number of estimated metastatic lymph nodes prior to NACT [23, 24, 36]. However, as this demonstrates, the focus has been on the number of lymph nodes showing a response to treatment, or not, whilst the features of that potential response have been presumed to be the same as those seen at the primary site.

## Features supportive of response

**Figure 1:**
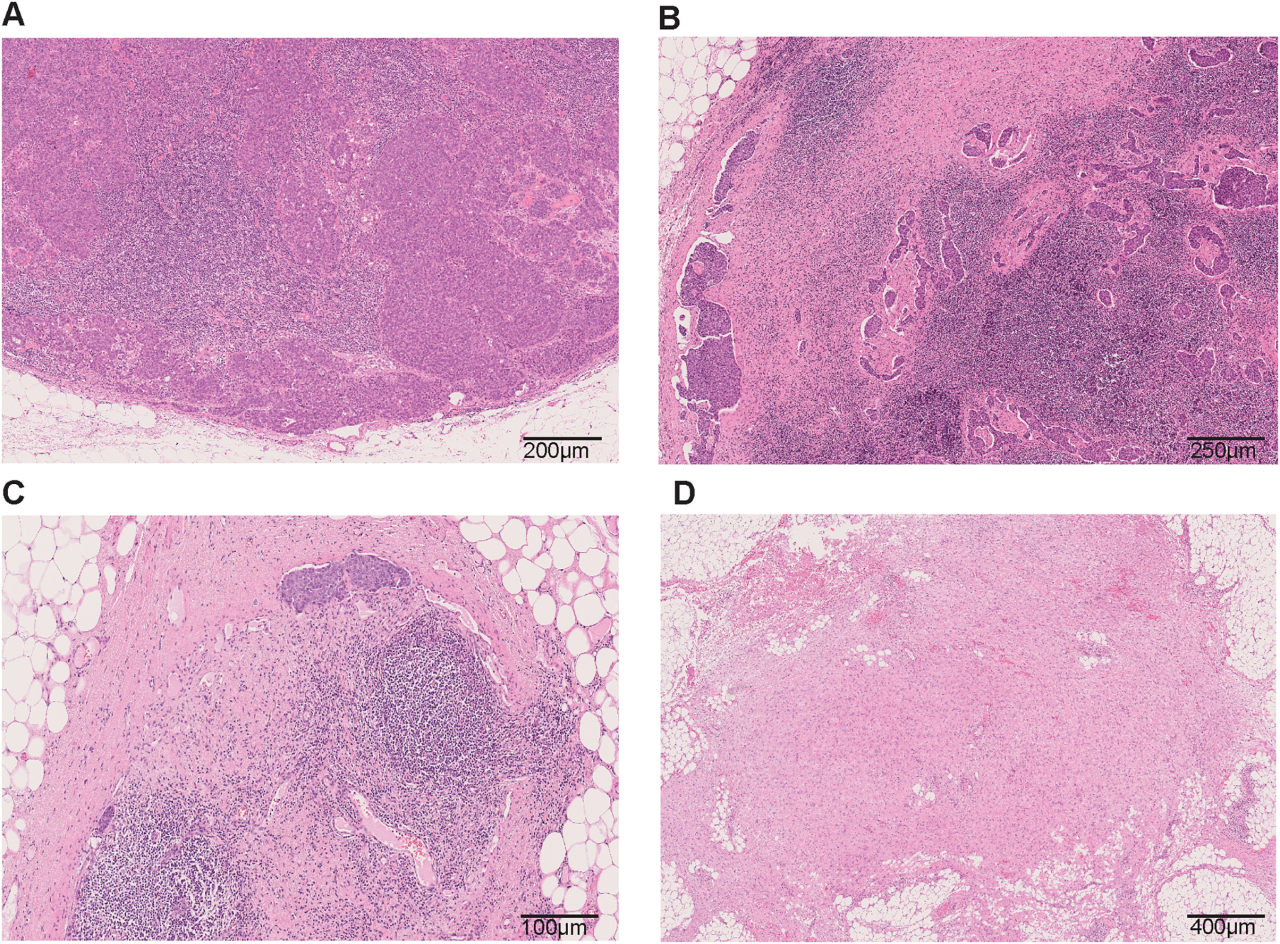
Histology of response to neoadjuvant chemotherapy (NACT) in axillary lymph nodes. (A) No response present in axillary lymph node after NACT; tumour cells and lymphoid cells adjacent to one another with no evidence of tumour bed. (B, C) Partial response in axillary lymph nodes after NACT; mixture of viable tumour cells, fibrosis, and macrophages resulting in partial loss of lymph node architecture. (D) Complete response to NACT, with obliteration of lymph node architecture and replacement by fibrosis.

The cellular and subcellular level changes at the primary breast tumour site have been reviewed elsewhere [37, 38, 39]. Histologically, in the breast, the tumour response bed is typically identified by loose, oedematous reactive stroma associated with a mixed inflammatory infiltrate, often including lipid‐ or haemosiderin‐laden macrophages, lymphocytes, and plasma cells [9]. As in other adenocarcinomas, mucin pools can be seen. Necrosis can be present. Other changes noted include neovascularisation, cholesterol clefts, and calcification (Figure 1).

However, these histological features are not unique to NACT. For example, reactive fibrotic stroma is an evolutionary conserved response to tissue injury seen in physiological and disease processes. It is a multicellular chronic process involving inflammatory reactions, angiogenesis, and fibroblast deposition of collagen and extracellular matrix [40].

Publications on lymph node response to NACT have examined different combinations of the histological features seen at the primary site to assess if there is a response present in the lymph nodes [23, 24, 41, 42]. The only lymph node‐specific change that may be seen is thickening of the capsule [41, 43]. A limitation of this approach is that lymph nodes have a unique architecture and function that differ from the primary site. Furthermore, lymph nodes which have never had metastases present may show histological features of relevance and prognosis, as is seen in patients receiving adjuvant chemotherapy; for example, the presence and number of germinal centres in involved and uninvolved lymph nodes have been shown to be prognostically relevant in TNBC patients [44, 45]; the significance of these features in lymph nodes following NACT is currently unknown.

**Figure 2:**
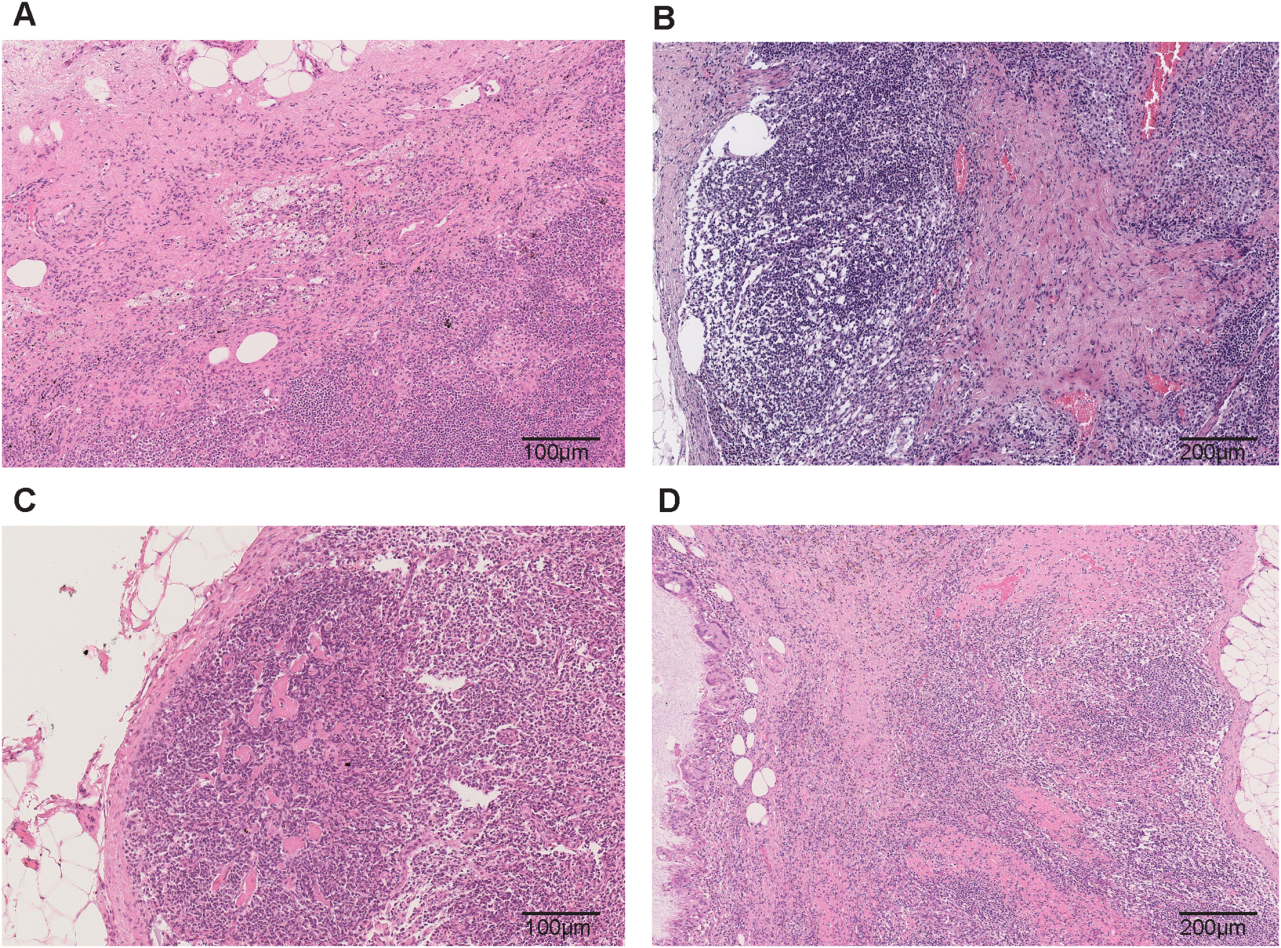
Mimics of response to neoadjuvant chemotherapy (NACT). (A) Response to NACT with regression of metastatic tumour cells and presence of lipid‐ and haemosiderin‐laden macrophages and cellular fibrosis adjacent to capsule. (B) Likely pre‐existing (not related to NACT) parenchymal fibrosis in a negative lymph node. (C) Focal hyalinised paucicellular fibrosis interpreted as pre‐existing within a negative node. (D) Marker clip site (left) seen as a cystic space lined by multinucleated macrophages peripherally and mucous‐like material within. An area showing NACT response with fibrosis, haemosiderin, and disruption of the lymph node architecture lies adjacent.

There are several mimics of response to NACT in the lymph node but there are no routinely available diagnostic tests that can be helpful. Previous biopsy site changes, previous surgery, and marker clip site can all appear with aggregates of macrophages, haemosiderin, varying degrees of fibrosis, and calcification [46, 47] (Figure 2). Furthermore, these can lie immediately adjacent to true treatment response and confound the interpretation and amount of response being reported. Multinucleated giant cells at previous surgical sites can rarely be mistaken for atypical epithelial cells and may require cytokeratin immunohistochemistry confirmation.

Within the lymph node, focal hyalinised fibrosis can potentially be long standing and unrelated to treatment. Lymph nodes react to numerous inflammatory triggers throughout life and occasionally this results in overproduction of extracellular matrix by fibroblast reticular cells. This can be present in the adjuvant treatment setting [32], i.e. unrelated to prior receipt of chemotherapy. Fibrosis has also been described as part of the degenerative changes with ageing and as a contributor to immunosenescence [48]. More rarely, systemic rheumatological diseases, HIV, and post‐granulomatous infections can also be a cause of fibrosis [49, 50, 51]. Some clues to the fibrosis being a result of response to chemotherapy rather than chronic is it being wedge‐shaped at the periphery and associated with a thickened capsule.

## Differences to the primary tumour

The degree of concordance between response in the primary breast tumour and in the lymph nodes is variable (14–89%) in the literature [19, 23, 42, 52, 53]. This might partially be explained by the tendency to report only concordance in pCR (or not) rather than a more subtle comparison of degrees of response, in conjunction with differing cohorts of patients and varying chemotherapy regimens. With the axilla more frequently achieving pCR than the breast, research into reasons for the discordance is lacking but clearly there are differences in the tumour microenvironment in the breast and lymph nodes, including metabolic factors, intrinsic tumour heterogeneity, and delivery and exposure to the cytotoxic agents.

The structure of the lymph node reflects its function to develop and orchestrate an adaptive immune response. With its cellular composition being vastly different to the primary site, including follicles of highly proliferating cells, the impact of NACT on the wider lymph node function is unknown. Furthermore, although the morphology of response is histologically interpreted similarly between the primary cancer and any lymph node metastasis, due to a lack of research into lymph node‐specific response, identifying features that are potentially more frequent, or of more prognostic relevance, at either site is not currently possible. One study of 85 breast cancer patients descriptively compared the frequency of pathological features of response between the breast and lymph nodes [42]; loose fibroelastosis was most often seen in both but with higher frequency in the breast. Dense homogeneous fibrosis, macrophage aggregates, and haemosiderin‐laden macrophages were also described as being present in both sites and, except for dense homogeneous fibrosis, being more frequent in the breast.

To add further to the complexity, there can be multiple lymph nodes with different degrees and patterns of response. Identifying these and potentially assimilating them into an index or score of prognostic or predictive value has not been attempted. The RCB includes measurement of the largest residual metastases but includes no assessment of other changes in any involved or uninvolved nodes. Other systems consider nodes together despite mixed patterns of response and the presence of viable tumour, and for the node staging in TNM, it is purely the number with metastases remaining and the size of the largest contiguous metastatic focus that are used for classification [14, 26].

## Quantification and reproducibility

One area of subjectivity is when one or more islands of focal intranodal fibrosis are present and when there is uncertainty as to whether it is a response to therapy or pre‐existing fibrosis. Such fibrosis is often free of tumour cells and macrophages, and is frequently paucicellular. No markers are available to help distinguish these small areas as pre‐existing or a response to NACT, and in general, pathologists will only classify an island as indicating response of previous metastasis when there are additional features such as additional reactive changes and, in particular a wedge‐shaped outline that extends from the capsule rather than being rounded and parenchymal.

Currently, methods to assess the degree of response to NACT are semi‐quantitative with potential issues of intra‐ and inter‐pathologist agreement. This has not been widely studied, although the overall RCB is reported to have good concordance [54, 55]. Documenting only the number of lymph nodes that remain positive is likely to result in high agreement. However, residual tumour could potentially be expressed as a percentage of the response bed or the lymph node area, which might provide more information. As with other semi‐quantitative histological assessments, for example stromal tumour‐infiltrating lymphocytes (TILs), this will take training and benchmarking with others to improve reproducibility [56]. Discrete categories for classifications, e.g. more or less than 50%, would also give better reproducibility but with the loss of potentially clinically relevant fine detail.

The digitalisation of pathology workflows facilitates the measuring of disease processes as well as the ability to integrate tumour detection software [57]. The potential for artificial intelligence (AI) to identify and measure different cellular components and infer prognostic and predictive biomarkers from the H&E sections could in time aid a more uniform approach to reporting response. Fine‐tuning of foundation models to this task‐specific problem, whilst maintaining high accuracy and generalizability, will allow for development in this field [58].

## Is a more detailed approach worth investigating?

Classifications evolve with new understanding and technical advancements. However, some of the studies investigating axillary lymph node response to NACT and improved prognostication are from more than 20 years ago [13, 20, 23, 59]. In 2003, Newman *et al* recorded histological evidence of response in lymph nodes, and identified that breast cancer patients with features of response had an intermediate survival between those with node‐positive and node‐negative disease [23]. More recently, Barrio *et al* described that 94% of a biopsy‐proven node‐positive breast cancer cohort showed some degree of treatment response in their lymph nodes [41]. Whilst radiological and pathological concordance of pCR is still not optimal and appears significantly impacted by subtype, with 67% sensitivity and 85% specificity for TNBC compared with 60% sensitivity and 74% specificity in HR‐positive/HER2‐positive breast cancer, pathological assessment remains a critical component for patient prognostication [4].

Limited research in breast, lung, oesophageal, and gastric cancers has begun to investigate different approaches to recording lymph node response after NACT, some of which has reported a prognostic benefit if this is seen, irrespective of the primary tumour characteristics and response and patient demographics [24, 60, 61, 62, 63, 64, 65, 66] (Table 1).

**Table 1:** Summary of published work investigating novel lymph node assessment and classification following NACT.

Cancer type	Year	No. of patients	Lymph node classification	Classification definition	Further classification
Breast [24]	2021	563	Grade 0	No metastasis without regression	
			Grade 1	No metastasis with complete regression (complete response)	
			Grade 2	Metastasis but complete regression in some LNs (partial response)	
			Grade 3	Metastasis and no complete regression in LNs (no response)	
Breast [60]	2019	72	Nodal pathological complete response	Absence of any invasive tumour cells (including isolated tumour cells) with evidence of treatment effect	
			Nodal partial response	Metastasis responding to a variable extent	
			Nodal mixed response	Metastasis with no and/or partial response and with presence of pathologically complete response	
			Nodal stable disease	Metastasis without evidence of tumour regression	
Lung [61]	2021	336	Low residual viable tumour	Less than or equal to 8% viable tumour in the tumour bed (necrosis and stromal fibrosis)	
			High residual viable tumour	More than 8% viable tumour in the tumour bed (necrosis and stromal fibrosis)	
Lung [62]	2021	75	Major pathological response positive	Less than or equal to 70% viable tumour in the tumour bed area (necrosis, fibrosis, inflammation)	
			Major pathological response negative	More than 70% viable tumour in the tumour bed area (necrosis, fibrosis, inflammation)	
Oesophageal [63]	2023	763	Lymph node regression score 1 (LNRS 1)	Regression only (complete response)	Complete response
			Lymph node regression score 2 (LNRS 2)	<10% viable tumour	Partial response
			Lymph node regression score 3 (LNRS 3)	10–50% viable tumour	Partial response
			Lymph node regression score 4 (LNRS 4)	>50% viable tumour	No response
			Lymph node regression score 5 (LNRS 5)	Viable tumour with no evidence of response	No response
			Negative lymph node	Without viable tumour or evidence of regression	
Oesophago‐gastric [64]	2023	1,619	Lymph node regression present	Presence of fibrosis, mucin lakes without viable tumour cells, large regions of foamy macrophages, and areas of necrosis with or without tumour cells	
			Lymph node regression absent	Absence of fibrosis, mucin lakes without viable tumour cells, large regions of foamy macrophages, and areas of necrosis with or without tumour cells	
Gastric [65]	2023	160	Nodal status positive	Tumour cells infiltrate lymph node	
			Nodal status negative	No evidence of tumour cells infiltrating lymph node	
Oesophageal [66]	2015	90	Negative lymph node	Without evidence of metastatic disease	
			Treatment response nodes	With evidence of prior cancer involvement but no currently viable cancer cells	
			Positive lymph nodes	Involved with metastases	

A four‐tier lymph node regression grade for all lymph nodes was devised by investigating 563 HR‐positive and HR‐negative invasive breast cancer patients [24]. The authors counted lymph nodes showing complete tumour regression (grade 1) to estimate the pretreatment status and calculate the ratio of lymph nodes with residual disease after chemotherapy to estimated pretreatment metastatic lymph node number. In multivariate analysis, their lymph node regression score was found to be an independent prognostic factor for disease‐free survival (DFS). Nevertheless, in patients with no evidence of metastases (ypN0), those with three or more lymph nodes showing complete response (grade 1) had a poorer DFS compared with those with complete response in fewer than three lymph nodes, highlighting that an index incorporating pretreatment disease burden along with response in the lymph nodes may further stratify patients.

Another approach used a percentage cut‐off response compared with viable tumour area; for example, 50% is used in the Sataloff four‐tier classification [67]. This was applied to analyse the lymph node response in 72 node‐positive breast cancer patients [60]. From that, they devised a three‐tier classification: nodal pathological complete response, nodal partial or mixed response, and nodal stable disease. The study's focus was the differing frequencies of response according to biological subtype of invasive breast cancers rather than survival analysis. HR‐positive/HER2‐negative breast cancer patients had the highest rate of nodal partial or mixed response, which was defined by the authors as heterogeneous response and could include the simultaneous presence of pathological complete response, partial response to metastases, and no response to metastases.

Two studies in lung cancer patients also examined the value of calculating the percentage of viable tumour. Liu *et al* recorded necrosis, stromal fibrosis, and viable tumour to the nearest 5% of the lymph node tumour bed of 336 non‐small cell lung cancer patients [61]. The optimal cut‐off for distinguishing a difference in overall survival and DFS was an 8% residual viable tumour within the tumour bed; patients with more than 8% residual viable tumour in multivariate analysis had a significantly poorer DFS. Pataer *et al* dichotomised a cohort of 75 node‐positive lung cancer patients according to the presence of more or less than 70% viable tumour cells in the tumour bed area within their lymph nodes [62]. Patients with 70% or less viable disease were defined as having a major pathological response, which was significantly associated with an improved overall survival. The striking difference between the cut‐offs used (8% versus 70% viable tumour percentage), even for one carcinoma type, is noteworthy and underscores that determination of a percentage viable tumour remaining in a lymph node that is of potential clinical benefit would take numerous studies and cohorts to both establish and validate the optimal cut‐point to be used.

In upper gastrointestinal (UGI) carcinomas, a range of approaches have been published. A multicentre study of 763 oesophageal adenocarcinoma patients reported the area of residual viable tumour to total area of tumour response bed [63]. A total of 17,930 lymph nodes were classified into six groups: negative nodes (without viable tumour or evidence of response); those with complete response; those with residual tumour thresholds of <10%, 10–50%, and >50%; and viable tumour with no evidence of response. Subsequently, at a patient level, different classifications were evaluated to predict survival. The authors found that classification by the best lymph node response and by condensing the six‐group classification into a four‐group classification (negative lymph nodes, complete response, partial response, and no response) was superior to other systems and demonstrated a significantly improved inter‐observer agreement. Furthermore, this new classification provided information beyond the currently used TNM staging and primary tumour regression score.

Two studies in UGI cohorts documented the presence or absence of regression and compared this with pathological TNM staging for predicting overall survival [64, 65]. However, neither study found an improved prognostication beyond the TNM system. Another study focused only on lymph nodes showing evidence of prior cancer involvement but no currently viable cancer cells (treatment‐response nodes) following NACT, and preoperative radiotherapy in a subset, and how incorporating these into the lymph node staging might affect survival predictions [66]. In patients classified as ypN0 or ypN1 (68% of the cohort), those with treatment‐response nodes had a significantly poorer survival compared with those lacking treatment‐response nodes, even when accounting for patient age and the AJCC seventh edition oesophageal cancer staging system.

## Future directions and conclusion

Lymph nodes are critical for generating a highly specific adaptive anti‐tumour immune response. NACT targets metastases within them to differing degrees. As at the primary site, response between patients and between different foci of disease within an individual may be variable but understanding this, and specifically anticipating those who will respond, is an active research area. Knowledge is still grounded in NACT targeting highly proliferating cells, despite many years of use, and there are currently too few studies to dissect out the molecular characteristics of the lymph node response [68, 69]. Future research, leveraging and combining advances in multiplex immunohistochemistry and immunofluorescence, spatial transcriptomics, and even three‐dimensional histology, could unlock the molecular drivers of the tumour, stromal, and immune response [70, 71, 72].

Immunotherapy is now the standard of care for some patients, alongside NACT, and the implications of immune cross‐talk between the primary site and the lymph nodes are gaining attention and highlighting the potential need for lymph nodes to be present for immunotherapy to be most effective [73, 74]. However, the histological response to NACT plus immunotherapy is not yet described at a morphological level and, at present, such cases are reported histopathologically in the same way as those in receipt of NACT alone.

**Figure 3:**
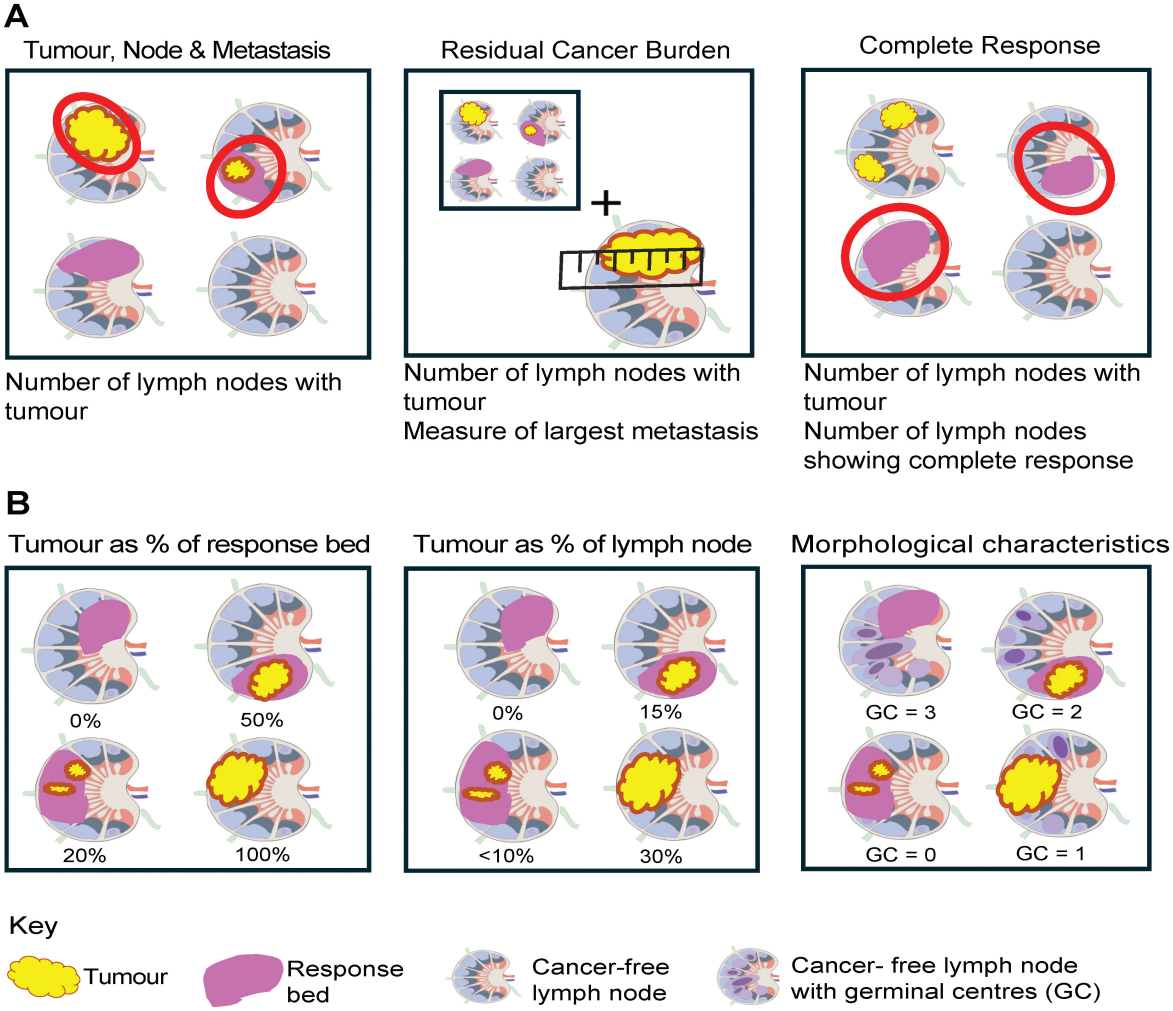
Lymph node assessment. (A) Current axillary lymph node assessment after neoadjuvant chemotherapy (NACT). The tumour, node, metastasis (TNM) staging system reports the number of lymph nodes that have metastases present. The residual cancer burden (RCB) score includes the number of lymph nodes that have metastases present and the measurement of the largest metastasis. Reporting the number of lymph nodes with complete response (no viable tumour cells) in addition to the number of lymph nodes that have metastases present is recommended by the International Collaboration on Cancer Reporting (ICCR). (B) Potential future axillary lymph node assessment after neoadjuvant chemotherapy (NACT). Viable tumour present after NACT could be reported as either a percentage of the response bed area or a percentage of the lymph node section area. Morphological characteristic such as germinal centres (GCs) of involved and uninvolved lymph nodes could be reported.

Any system of histological assessment of the lymph nodes needs to be reproducible and meaningful to patients’ prognosis and ongoing management. Across the limited papers currently evaluating a more granular lymph node assessment, i.e. beyond the number of positive nodes and the size of the largest metastasis, it appears that incorporating nodes with complete response (that currently would be simply categorised as negative) into a classification system could have benefit. Furthermore, documentation of the percentage of viable tumour remaining within the nodal tumour bed after NACT and potentially other response changes within a node could valuably be explored in breast cancers, as these have been shown in some lung and UGI cancer cohorts to be significantly linked to outcome (Figure 3).

At present, there is a scarcity of published work specific to the axillary response in breast cancer patients receiving NACT, and improved prognostic stratification based on this would be valuable. Potentially, it could aid the tailoring of management decisions around adjuvant therapy and surgical decisions for patients with residual disease, especially those with isolated tumour cells. Recently published clinical trials demonstrate an appetite for investigating how modifications to adjuvant approaches based on the post‐NACT nodal status may impact survival [75, 76].

No published research has, as yet, considered changes to the background lymph node architecture that represents crucial biological processes for immune response. Germinal centres and expanded sinuses are architectural changes observed that provide insight into ongoing immunological processes, such as antigen presentation and the creation of long‐lived memory B cells and plasma cells. Capturing the patterns of these in the adjuvant setting shows prognostic value, and assessment in the neoadjuvant setting warrants attention. In particular, machine learning may be capable of doing this task in a reproducible manner.

Prior to recommending a change to the reporting of axillary lymph nodes after receipt of NACT, whether that is a new classification, or suggesting routine reporting of a single specific histological feature, rigorous multicentre evaluation and validation would be required. Additionally, for ease of pathological reporting, a system that could be universally applied across biological subtypes would be beneficial.

Another consideration is the evolving surgical landscape, for example with targeted axillary dissections (TADs) increasing in patients with clinically good response to NACT instead of axillary clearance surgery. Nevertheless, there is a lack of standardisation in patient selection for this procedure and onward management following pathological assessment. A concern is that residual metastatic lymph nodes go undetected following TAD due to imaging fallibility and variable concordance between breast and nodal pCR, but this concern can be lessened when pCR is achieved in the breast as this is associated with higher rates of lymph node pCR [53].

In conclusion, present‐day assessment of axillary lymph nodes following NACT in breast cancer patients is entirely numerical and includes the number of nodes remaining involved and the size of the largest metastasis. Whilst this appears standardised and uniform, a potentially more nuanced approach, accounting for the morphological changes in nodes with metastasis, in nodes with complete response, and in those remaining uninvolved, may be clinically relevant for guiding management decisions in the adjuvant setting, despite potential issues with reproducibility and the increased workload of such assessment. Furthermore, the axillary lymph nodes, as the first site of metastasis and as a hub for a highly specialised adaptive immune response, offer unparalleled opportunities to explore tumour biology and anti‐tumour immunity. These insights will be particularly critical in the current era of advancing immunotherapy for breast cancer patients.

## Author contributions statement

LR, AG and SP were responsible for conceptualisation. LR wrote the original draft. LR, EP, AG and SP edited the review. All authors agreed with the final manuscript.

## Data Availability

Data sharing not applicable to this article as no datasets were generated or analysed during the current study.
